# *CLEC16A* regulates splenocyte and NK cell function in part through MEK signaling

**DOI:** 10.1371/journal.pone.0203952

**Published:** 2018-09-18

**Authors:** Rahul Pandey, Marina Bakay, Heather S. Hain, Bryan Strenkowski, Barakat Z. B. Elsaqa, Jeffrey D. Roizen, Jake A. Kushner, Jordan S. Orange, Hakon Hakonarson

**Affiliations:** 1 The Center for Applied Genomics, The Children’s Hospital of Philadelphia, Philadelphia, PA, United States of America; 2 Department of Medicine, Jordan University of Science and Technology, Irbid, Jordan; 3 Division of Endocrinology and Diabetes Children’s Hospital of Philadelphia, Philadelphia, PA, United States of America; 4 Section of Pediatric Diabetes and Endocrinology, Department of Pediatric Medicine, Endocrine-Metabolism, Texas Children’s Hospital, Houston, TX, United States of America; 5 Section of Immunology, Allergy, and Rheumatology, Department of Pediatric Medicine, Texas Children’s Hospital, Houston, TX, United States of America; 6 Department of Pediatrics, the Perelman School of Medicine, University of Pennsylvania, Philadelphia, PA, United States of America; Univerzitet u Beogradu, SERBIA

## Abstract

*CLEC16A* is implicated in multiple autoimmune diseases. We generated *Clec16a* inducible knockout (KO) mice to examine the functional link between *CLEC16A* auto-inflammation and autoimmunity. *Clec16a* KO mice exhibited weight loss and thymic and splenic atrophy. Mitochondrial potential was lowered in KO mice splenocytes resulting in aggregation of unhealthy mitochondria in B, T, and NK cells. In *Clec16a* KO mice we detected disrupted mitophagy in splenic B and T cells. NK cells from *Clec16a* KO mice exhibited increased cytotoxicity. Incomplete mitophagy was attenuated with PI3K and/or MEK inhibition in *Clec16a* KO mice. Our results demonstrate a functional link between *CLEC16A* and disrupted mitophagy in immune cells and show that incomplete mitophagy predisposes the KO mice to inflammation. Taken together, loss of function variants in *CLEC16A* that are associated with decreased CLEC16A expression levels may contribute to inflammation in autoimmunity through disrupted mitophagy. Drugs modulating mitophagy reverse the process and may be effective in treating and preventing autoimmunity in individuals with risk associated *CLEC16A* variants.

## Introduction

Genome-wide association studies (GWAS) have consistently identified associations between single nucleotide polymorphisms (SNPs) in the 16p13 locus harboring the C-type lectin-like domain family 16A (*CLEC16*A) gene and type-1 diabetes (T1D)[1–5]. *CLEC16A* locus polymorphisms have also been associated with the susceptibility to several other autoimmune diseases, including multiple sclerosis [6, 7], primary adrenal insufficiency [8], Crohn’s disease[8, 9], primary biliary cirrhosis[10], juvenile idiopathic arthritis[11, 12], rheumatoid arthritis[12] and alopecia areata[13], suggesting that *CLEC16A* could be a master regulator of aberrant immune responses in autoimmunity. Despite the strong associations of *CLEC16A* with numerous autoimmune and inflammatory disorders little is known about the function of CLEC16A or its role in disease pathogenesis. No study to date has investigated the possible connection of CLEC16A’s role in immune cells.

The first evidence of CLEC16A function came from studies on its drosophila ortholog-Ema, an endosomal membrane protein required for endosomal trafficking and maturation. Human CLEC16A expression was shown to rescue the drosophila null mutant demonstrating conserved protein function across species[14]. Ema is also required for normal autophagosomal growth and autophagy[15]. We recently showed that loss of Clec16a leads to an increase in Parkin, the master regulator of mitophagy, in mice with pancreas-specific deletion. Islets of these mice had abnormal mitochondria with reduced oxygen consumption and ATP concentration. Patients with the *CLEC16A* T1D risk variant, rs12708716-G, have reduced expression of CLEC16A in islets and attenuated insulin secretion[16], providing further evidence that CLEC16A could control β-cell function and contribute to diabetes risk through complex interactions between resident pancreatic cells, NK cells and potentially other immune cells where *CLEC16A* is known to play a key role[17]. Likewise, a role of CLEC16A in autophagy and neurologic diseases was reported in another independent mutant mouse model of *Clec16a*[18]. Thus, *CLEC16A* is becoming an attractive candidate for functional studies to explore the pathogenic mechanisms and therapeutic options through interventions at the protein level of CLEC16A in autoimmune diseases.

Given the association of *CLEC16A* to several autoimmune disorders we sought to understand the role of *CLEC16A* in immune cells. Mitochondrial dysfunction may contribute to the pathogenesis of autoimmune disease. Autoimmune diseases develop as a consequence of a synergistic combination of genetic predisposition, largely unknown environmental triggers, and immunologic events. We hypothesized that reduced expression of CLEC16A leads to disrupted mitophagy. In this study we used our novel whole body inducible *Clec16a* knockout model (UBC-*Cre-Clec16a*^*loxP*^) along with autophagy pathway inhibitors to delineate the role of CLEC16A in mitophagy in immune cells contributing to immune dysregulation. We demonstrate that reduction in CLEC16A is associated with disrupted mitophagy and defective mitochondria, effects that were attenuated by PI3K and/or MEK inhibitors.

## Materials and methods

### UBC-*Cre-Clec16a*^loxP/loxP^ mice

The Institutional Animal Care and Use Committee (IACUC) of the Children’s Hospital of Philadelphia approved all animal studies. All methods were performed in accordance with the IACUC guidelines and regulations.

Mice were group-housed on an individually-ventilated cage rack system on a 12:12 light: dark cycle. Mice were fed standard rodent chow and water *ad libitum*.

*Clec16a*^loxP^ mice were generated by flanking exon 3 (Ozgene). Mice with targeted insertion in the *Clec16a* gene were crossed to the Flpo Deleter line (mouse Strain: 129S4/SvJae-Gt (ROSA) 26Sortm2(FLP*) Sor/J; The Jackson Laboratory) to achieve deletion of the FRT-flanked Neomycin cassette. *Clec16a*^loxP^ mice were mated to UBC-*Cre-ERT2* mice (inducible cre recombinase driven by the human ubiquitin C promoter) obtained from The Jackson Laboratory to generate UBC-*Cre-Clec16a*^loxP^ mice. Mice were kept on mixed background C57BL/6-129S1.

Ten-week-old UBC-*Cre-Clec16a*^loxP/loxP^ mice were treated with tamoxifen (100 mg/kg/day) to induce knockout of *Clec16a* (*Clec16a* KO) or vehicle (10%: ethanol: 90% corn oil) for a control group by gavage at 24-hour intervals for five consecutive days. *Clec16a*^loxP/loxP^ mice were administered tamoxifen or vehicle and used as control groups.

All inhibitors were purchased from Sigma-Aldrich (St. Louis, MO, USA) with purity of greater than 98% confirmed by HPLC. For inhibitor experiments, ten-weeks-old UBC-*Cre*-*Clec16a*^loxP^ mice were treated with tamoxifen (100 mg/kg/day) or vehicle (10%: ethanol: 90% corn oil) for control group by gavage (10 ml/kg) and vehicle or inhibitor (i.p., 10 ml/kg) at 24-hour intervals for five consecutive days. Vehicle (specific for inhibitor) or inhibitor were injected 30 minutes prior to either vehicle or tamoxifen gavage. Vehicle for U0126 (10 mg/kg) and Wortmannin (2 mg/kg) was 1% DMSO: 10% Cremaphor: 89% of 0.09% saline and for Bafilomycin A1 (1 mg/kg) was 1% DMSO: 99% of 0.09% saline. Stock solutions were created for each compound and frozen. Fresh compound was made daily prior to injection. All animals were sacrificed according to humane endpoint or four weeks after initiation of the experiment.

### Quantitative real-time PCR

Total RNA was isolated with Trizol, purified using the RNAeasy Mini Kit (Qiagen) and converted to cDNA by High Capacity RNA-to-cDNA Kit (Applied Biosystems). Human and murine CLEC16A RNA transcripts and control genes (β-Actin and HRPT1) were measured by real time PCR on a ViiA™ 7 Real Time PCR. Triplicates were used for all the samples. PCR runs were performed on ViiA™ 7 Real Time PCR System using ViiA7 RUO software v1.2.2 (Life Technologies).

### Transmission electron microscopy

Purified B, T, and NK cells were prepared for transmission electron microscopy (TEM) as previously reported to analyze mitochondrial defects[19]. Briefly, cells were washed with pre-warmed PBS twice, and fixed in 2% glutaraldehyde in PBS for 60 minutes. Then, cells were washed with PBS 3 times and post fixed with 1% osmium tetroxide in PBS for 1 hour. Cells were rinsed 3 times with distilled water and were further stained with 1% tannic acid for 1 hour and then infiltrated and embedded in Epon resin. Ultrathin sections of 70 nm were generated with an ultramicrotome (Ultracut 7, Leica Microsystems) and post-stained with 2% aqueous uranyl acetate and Reynold’s lead citrate for 10 minutes each. Samples were examined with a JEOL 2100 TEM at an accelerating voltage of 200 kV.

### Cell cultures

The YAC-1 target was grown at 37°C in 5% CO_2_ in complete RPMI medium (Gibco). Complete’ indicates supplementation with 10% FBS, L-glutamine, nonessential amino acids, sodium pyruvate, HEPES and penicillin-streptomycin (all from Gibco). Murine splenic NK, T and B cell suspensions were negatively selected using EasySep Mouse NK, T and B cell Enrichment Kits, following the manufacturer’s instructions (StemCell).

### Flow cytometry

To assess mitochondria membrane potential in control and knockout, rmIL-15 activated splenocytes, cells were stained with MitoTraker® Deep Red FM (31.3nM) and analyzed by FACS. Cell-associated fluorescence was assessed with FACSCalibur flow cytometer (BD Pharmingen) and analyzed with FlowJo software.

### NK cell cytotoxicity assays

Splenocytes from the control and *Clec16a* KO mice where specified were used as effector against ^51^Cr-labeled YAC-1 targets at effector-to target cell (E: T) ratios of 50:1, 25:1, 12.5:1, 6.25:1, and 3.12:1. In-vitro IL-15 stimulation of murine splenocytes was performed by adding 100 ng/ml of rmIL-15 for 48hrs where indicated. All test conditions were performed in triplicate, and supernatants harvested after 4 hours of incubation at 37°C were evaluated for the presence of ^51^Cr using a Top Count XL counter and Lumiplate scintillation system (Beckman Coulter). Cytotoxicity was defined as the lysis of YAC-1 target cells. Maximal release was determined by counts obtained after the incubation of target cells in 0.5% NP40.

### Western blot

Briefly, lysis was performed with NP40 lysis buffer. The lysates were electrophoresed on 4–12% NuPAGE Bis-Tris gels in MOPS SDS running buffer and transferred onto nitrocellulose membranes (Invitrogen) overnight. The membranes were blocked in 3% BSA and incubated with indicated primary antibodies where specified for: mouse CLEC16A, PINK1 (Abgent), GFP, Nrdp1 (Novus Biologicals), TOM20 (ProteinTech), Parkin, p62/SQSTM1, LC3 I/II, ATG16L1, cytochrome c, caspase-9, p-ERK1/2 and EK1/2 (Santa Cruz), cleaved Caspase-3, p-Akt (ser473), p-Akt (Ser308) and total Akt (Cell signaling). The membranes were washed and incubated with a respective mouse/rabbit secondary antibody and bound antibody was detected with WesternBright ECL kit (Advansta). Membranes were stripped and re-probed for β-actin as loading control.

### Interferon-γ (IFN-γ) ELISA

Human IFN-γ was detected using DouSet ELISA system (R&D System) as per manufactures instructions. EBV-immortalized lymphoblastoid cell lines generated from Type1 diabetes patients were activated with 100 ng/ml PMA plus 1 μg/ml ionomycin (PMA+I). Culture supernatants were collected after 48 hrs of induction. Supernatants were collected at indicated time points and IFN-γ was measured by DouSet ELISA system.

### Mouse Cytokine Array

The Proteome Profiler Mouse Cytokine Array Kit, Panel A (ARY006, R&D Systems) was used to determine systemic chemokine/cytokine profile. Briefly, plasma was diluted and mixed with a cocktail of biotinylated detection antibodies. The sample/antibody mixture was then incubated with the array membrane overnight at 4°C. The membranes were washed and incubated with streptavidin-horseradish peroxidase followed by chemiluminescent detection. The array data were quantitated to generate a protein profile, and results are presented as average signal (pixel density) of the pair of duplicate spots representing each cytokine or chemokine analyzed using Image-J software (NIH).

### Statistical analysis

All graphs denote mean values, and error bars represent the SE. Data were analyzed using unpaired Student’s t-test or factorial analysis of variance (ANOVA) as applicable using Prism 7 (GraphPad Software, Inc). Multiple sets of data were compared using one-way and two-way ANOVA Tukey’s multiple comparison test where appropriate. P values less than 0.05 were considered statistically significant.

## Results

**Fig 1:**
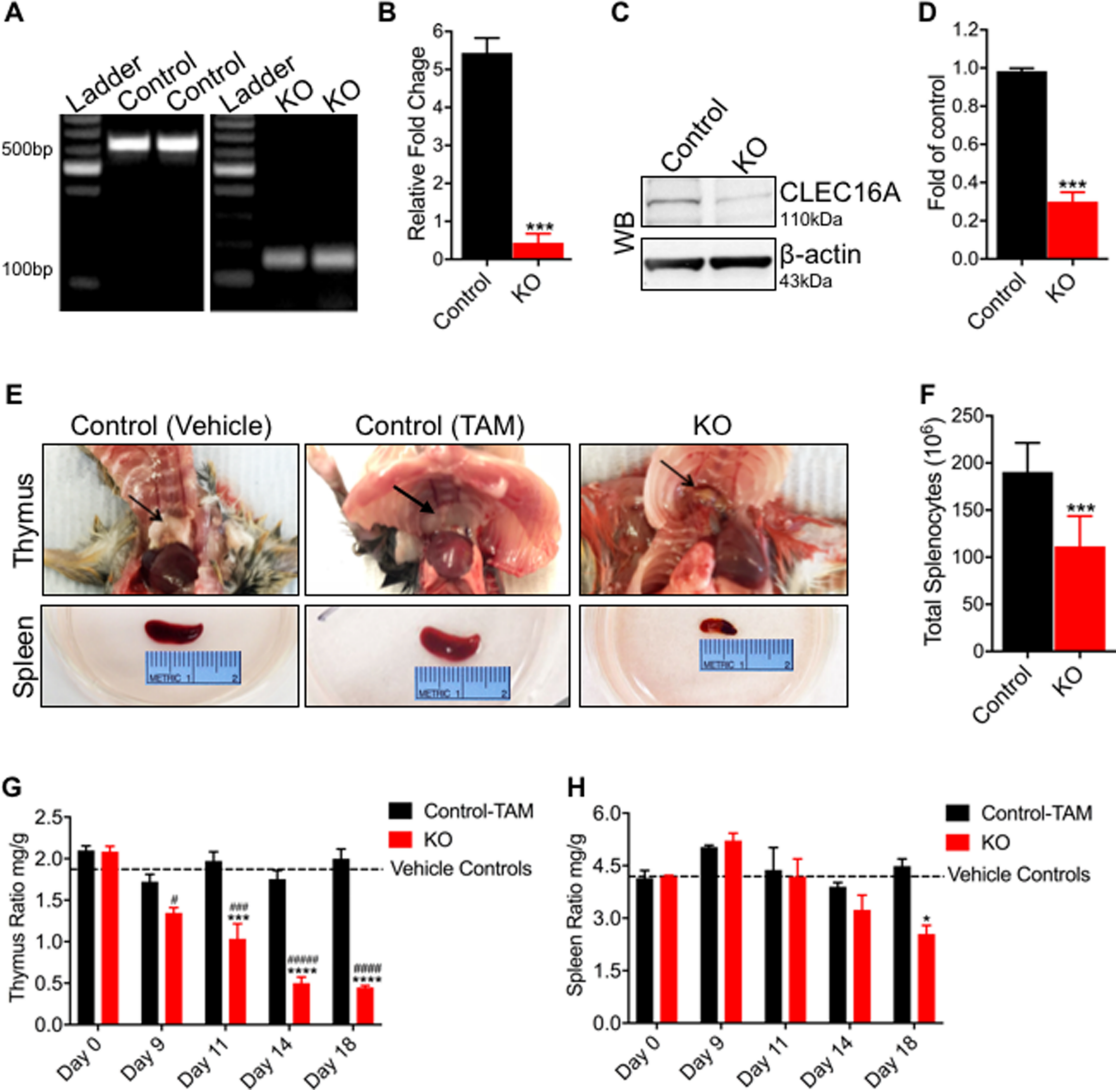
Characterization of *Clec16a* KO in tamoxifen-inducible UBC-*Cre-Clec16a*^loxP/loxP^ mice. (A) PCR analysis of genomic DNA isolated from whole blood of KO and control (vehicle and TAM treated) mice show PCR products of 618bp in control**s** and 146bp in KO, confirming a removal of *Clec16a* exon 3 in KO mice. (B) Relative *CLEC16A* mRNA expression by RT-PCR in murine splenocytes normalized to HPRT1. RT-PCR results show means±SE of three experiments. (C) Representative Western blot of CLEC16A expression in murine splenocytes. (D) Quantitation graph depicting CLEC16A protein expression (n = 3 repeats). (E) Reduction in total body weight of *Clec16a* KO mice. Multiple t tests were used to analyze data. Statistical significance was determined using the Holm-Sidak method. **P<0.001, ***P<0.001. (F) Thymus image from control (vehicle), control (TAM) and KO (top panel). Spleen image from control (vehicle), control (TAM) and KO mice (bottom panel). (G) Total splenocyte numbers from control and KO mice (n = 10). Significances of differences among groups were evaluated using an unpaired Student’s t-test ***P<0.001. (H) Thymus weight/body weight ratios for control (vehicle or TAM) and KO groups. (I) Spleen weight/body weight ratios for control (vehicle or TAM) and KO groups. Dashed line represents vehicle controls. Significances of differences were analyzed using multiple t-tests with discovery determined using the Two-stage linear step-up procedure of Benjamini, Krieger and Yekutieli, with Q = 1%. Computations assume that all rows are sample from populations with the same scatter (SD). Sidak's multiple comparison populations with the same scatter (SD)***P<0.001 and ****P<0.0001 between groups. Sidak's multiple comparisons test ^#^p<0.05, ^##^p<0.01, and ^###^p<0.0001 compared to Day 0 within groups test.

To investigate *CLEC16A* function, we generated inducible whole-body *Clec16a* knockout mice (UBC-*Cre-Clec16a*^loxP^). Adult mice (10 weeks old) were treated with tamoxifen to induce *Clec16a* Knockout shown in supplemental materials (S1 Fig). Control littermate mice received an equal volume of vehicle or tamoxifen. Recombination was confirmed by PCR using DNA isolated from whole blood (Fig 1A). Knockout was confirmed by RT-PCR on RNA (Fig 1B) and Western blot for CLEC16A protein expression from control and KO mice splenocytes (Fig 1C). The *Clec16a* KO mice exhibit significant reduction in body weight as early as day 9 compared to controls. The decrease became more significant over the course of the study (Fig 1E). In addition to weight loss, *Clec16a* KO mice exhibit atrophy of the thymus (top panel) and spleen (bottom panel) (Fig 1F) and significant reduction of total splenocyte numbers (Fig 1G). The thymus exhibited acute atrophy in KO mice. The *Clec16a* KO mice exhibited significant decrease in thymus weight and thymus/body weight ratios as early as day 9 compared to controls. The decrease became more significant over the length of the study (Fig 1H). Spleens showed a gradual regression in size. The *Clec16a* KO spleen weight/body weight ratio became significant by day 18 in comparison to controls (Fig 1I). Control vehicle and control-TAM littermates showed no atrophy of thymus and spleen.

**Fig 2:**
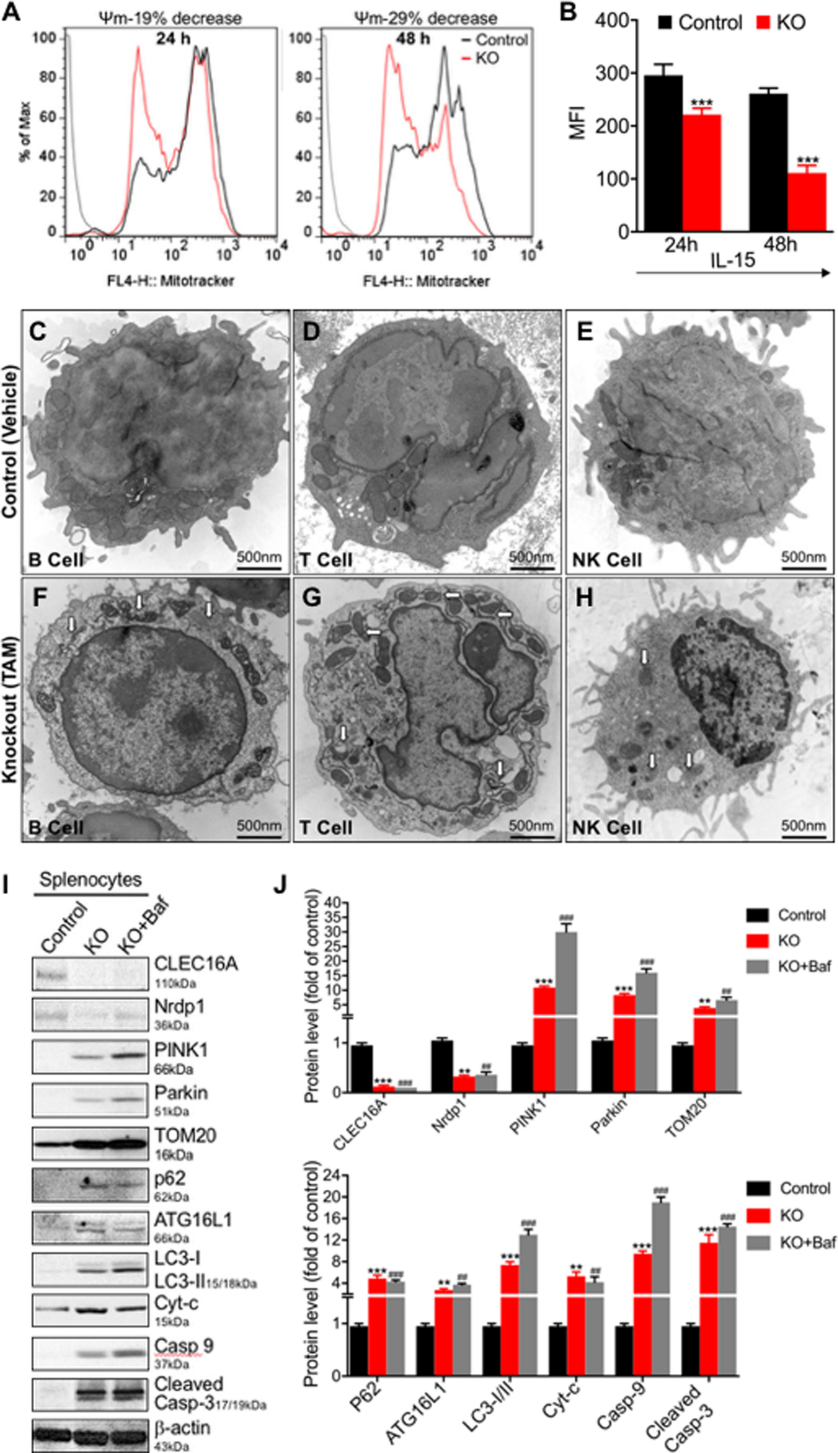
*Clec16a* knockout disrupts mitophagy *in-vivo* in response to lowered mitochondrial membrane potential resulting in aggregation of fragmented mitochondria in splenic immune cells. (A) The histogram depicts lowered mitochondrial membrane potential in splenocytes from *Clec16a* KO mice (n = 7) upon *Clec16a* KO in comparison to control-vehicle treated (n = 6) littermates using MitoTracker Deep Red FM dye in a flow-based assay. (B) Mitochondrial membrane potential depicted as change in mean fluorescence intensity (MF) for two time points, 24h and 48h. (C-H) Representative Transmission Electron Microscopy images of immune cells (splenic B, T and NK cells: control (Vehicle) (C-E) vs *Clec16a* KO (F-H). (I) Representative immune blot images depicting Nrdp1/PINK1/Parkin dependent incomplete mitophagy in *Clec16a* KO mice. (J) Quantitation graph depicting expression levels of CLEC16A, Nrdp1, PINK1, Parkin, p62, TOM20, ATG16L1, LC3I/II, Cytochrome-c, Caspase-9 and cleaved caspase-3 from control and KO±Bafilomycin treated mice normalized to β-actin. Data expressed as means±SE of three independent experiments. *P<0.05, **P<0.01, ***P<0.001, ^**#**^P<0.05, ^**##**^P<0.01, ^###^P<0.001. Significances of differences among groups were evaluated using unpaired Student’s t-test.

Since essentially no thymus was left in *Clec16a* KO mice, to examine the possibility of a mitochondrial defect we measured the mitochondria membrane potential (MMP) in splenocytes isolated from *Clec16a* KO mice and control littermates. Resting and cells activated with murine recombinant interleukin-15 were evaluated for change in mitochondrial membrane potential using flow cytometry. Control and *Clec16a* KO unstimulated splenocytes showed no significant difference (S2A and S2B Fig). At 24h post activation we observed a 19% decrease in the percentage of active mitochondria in activated KO mice splenocytes compared to activated controls. This difference increased 48h post activation, reaching 29% compared to activated controls. Lowering of mitochondrial membrane potential is depicted as change in mean fluorescence intensity post IL-15 activation (Fig 2A and 2B). *Clec16a* KO was confirmed in an immunoblot analysis shown in supplement materials (S2C Fig). We also measured levels of P62 and LC-3 I/II from resting and activated splenocytes lysates in an immunoblot analysis to assess autophagic flux. Resting *Clec16a* KO splenocytes showed significant increase in P62 and LC3-I/II expression in comparison to resting controls. With activation the levels of accumulated P62 and LC3 I/II further increased significantly in IL-15 activated splenocytes (S2D and S2E Fig). Activated control splenocytes showed no significant change for P62 and LC3-I/II. Thus, *Clec16a* KO activated splenocytes exhibit lowered mitochondrial membrane potential and defective autophagic flux.

We next performed Transmission Electron Microscopy (TEM) of splenic immune cells (B, T and NK cells) isolated from control and *Clec16a* KO mice to compare the mitochondrial morphology. *Clec16a* KO mice show dramatic qualitative changes in mitochondria and vacuolar morphology in immune cells (splenic B, T, and NK cells: control (Fig 2C–2E) vs *Clec16a* KO (Fig 2F–2H). Specifically, TEM images reveal changes in the mitochondrial membrane architecture and accumulation of disordered, disintegrated, elongated and fragmented mitochondria and vacuolated structures in the *Clec16a* KO mice in all immune cell types (B, T, and NK cells). Fragmented and elongated mitochondria with abnormal cristae, engulfed in vacuolar structures are clearly visible in zoomed in images of B (S3 Fig) and T cell (S4 Fig).

We next evaluated the whole splenocytes lysates from control (Vehicle) ±Bafilomycin and *Clec16a* KO±Bafilomycin treated mice in an immunoblot analysis for possible mitophagy/autophagy signaling defects. Bafilomycin A1, a selective inhibitor of the vacuolar-type H^+^-adenosine triphosphatase blocks autophagic degradation. We observed reduced Nrdp1 expression in *Clec16a* KO mice compared to controls (Fig 2I and 2J). As anticipated, *Clec16a* KO mice splenocyte lysates showed increased expression of PINK1, Parkin and TOM20 and further accumulation upon bafilomycin treatment. Vehicle treated splenocyte lysates showed barely detectable levels of PINK, Parkin and P62. We found significant increase in p62 and LC3-II expression in *Clec16a* KO compared to control. P62 is degraded by autophagy and inhibition of autophagy increases its abundance. Bafilomycin treatment did not significantly alter the P62 and ATG16L1 levels. The rise in LC3-II following Bafilomycin treatment was small, suggesting a defect in autophagic flux. In light of decreased splenocyte numbers, we hypothesized there was increased cell death in *Clec16a* KO mice spleen. To test that, we examined the relative abundance of cytochrome-c and caspase-9 in *Clec16a* KO mice splenocytes to determine if disrupted mitophagy contributed to cell death. We detected significant increase in both cytochrome-c and caspase-9. Further evaluation in immunoblot analysis revealed a significant increase in expression levels of cleaved caspase-3 in *Clec16a* KO mice in comparison to control littermates. Splenocyte lysates from Bafilomycin treated KO mice showed further increase in caspase-9 and cleaved caspase-3 (Fig 2I), providing evidence that the intrinsic pathway of cell death is activated in these settings. Control treated Bafilomycin splenic lysate showed significant increase in the expression of ATG16L1, P62 and LC3-I/II compared to untreated controls. Bafilomycin treatment in control mice did not alter the expression of CLEC16A, Nrdp1, PINK1, and Parkin (S2F and S2G Fig). Thus, *Clec16a* knockout lowers mitochondrial membrane potential and triggers defective mitophagy *in vivo*.

**Fig 3:**
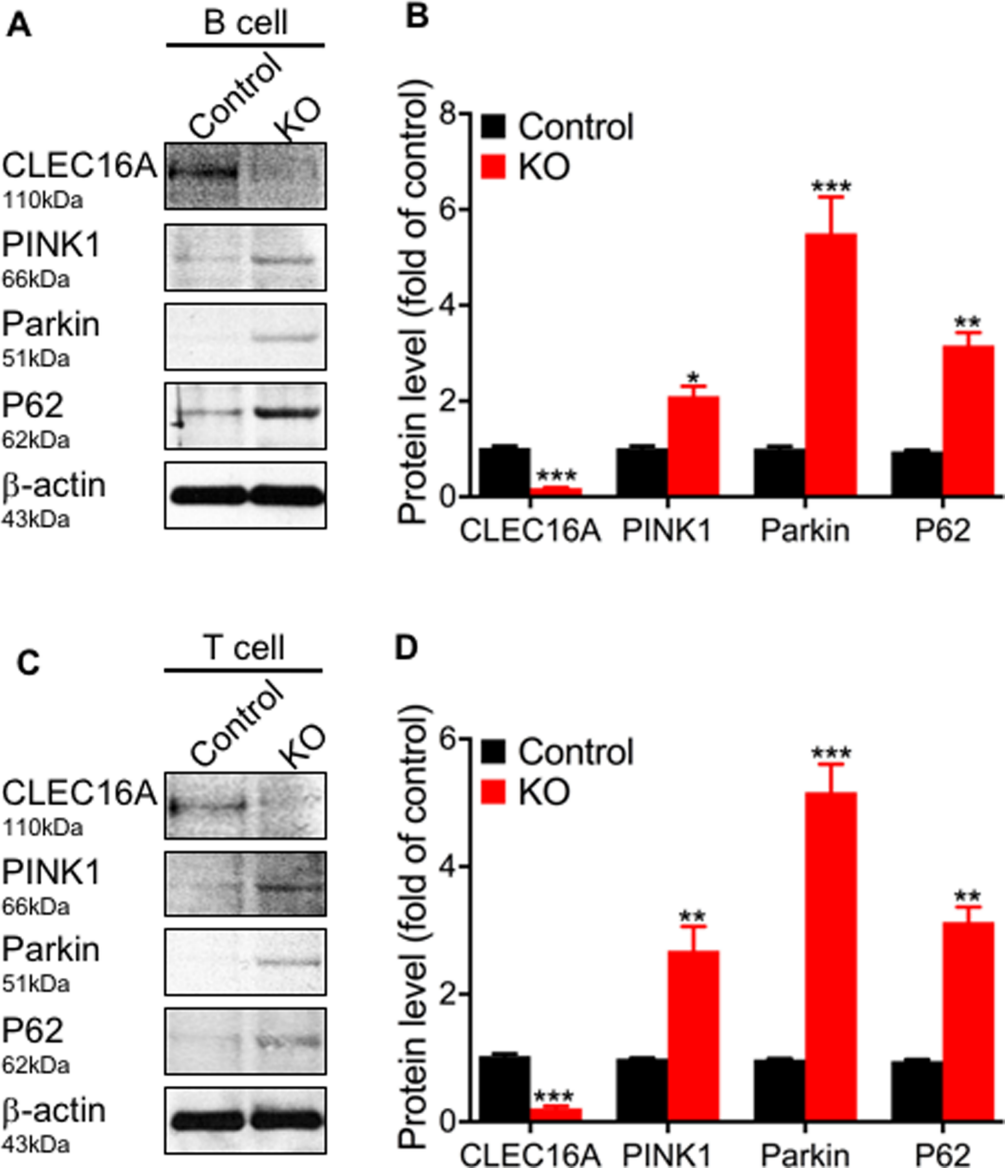
*Clec16a* knockout mice exhibit disrupted mitophagy in splenic B and T cells. (A) Representative Immunoblot analysis depicting expression levels of CLEC16A, PINK1, Parkin, p62, and β-actin in pure B cells isolated from control (Vehicle) and KO mice splenocytes. (B) Graph of quantitation depicting protein expression levels. Data expressed as means±SE of three independent experiments. Significances of differences among groups were evaluated using two-tailed unpaired Student’s t-test. (C) Representative Immunoblot depicting expression levels of CLEC16A, PINK1, Parkin, p62, and β-actin in pure T cells isolated from control and *Clec16a* KO mice splenocytes. (D) Graph of quantitation depicting protein expression levels. Data expressed as means±SE of three independent experiments. Membranes were striped and reprobed for β-actin as a loading control. *P<0.05, **P<0.01, ***P<0.001. Significances of differences among groups were evaluated using unpaired Student’s t-test.

We hypothesized that reduced expression of *Clec16a* in mouse leads to disrupted mitophagy via Nrdp1/PINK/Parkin pathway. To examine the signaling underlying the morphologic changes we observed in splenic immune cells of *Clec16a* KO by TEM, we performed immunoblot analysis. Since B-cells (60%) and T-cells (27%) contribute to the majority of the cell population of the spleen, we specifically evaluated pure B and T cells isolated from murine splenocytes (Fig 3A and 3C). Western blot analysis of purified B (Fig 3A and 3B) and T cell lysates (Fig 3C and 3D) in *Clec16a* KO showed significantly elevated levels of PINK1 and Parkin. Control lysates showed barely detectable levels of PINK and Parkin for both cell types. We observed significant increase and accumulation of P62 in B cells (Fig 3A and 3B) and T cells of KO mice (Fig 3C and 3D). Increased accumulation of P62 indicate that mitophagy is disrupted and incomplete in *Clec16a* KO B and T cells. We also evaluated the T cell subpopulations from control and *Clec16a* KO mice (S5A–S5C Fig). We observed a significant decrease for both CD4+ and CD8+ effector cell population (S5B and S5C Fig). However, naive and memory T cells in CD4+ and CD8^+^ cells remained unchanged. The decrease in effector population possibly could be attributed to increased apoptosis.

Since our whole body inducible *Clec16a* mice displayed thymic atrophy, and T cells play a key role in regulating the immune responses, to specifically address the role of *Clec16a* in T cells we generated CD4-*Cre Clec16a*^loxP^ mice. We followed CD4-*Cre Clec16a*^loxP^ mice up to 30 weeks of age and found no significant difference between the control and CD4 *Clec16a* KO mice thymic and splenic T cell subpopulation (S6A and S6B Fig). These results suggest that the CLEC16A loss alone in T cell is not enough to produce the phenotype shown in the whole-body inducible knockout.

**Fig 4:**
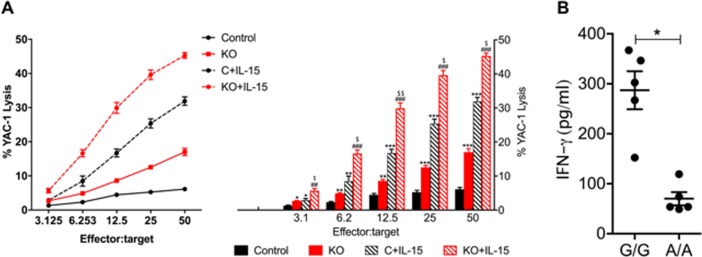
NK cytotoxicity of mouse splenic NK cells and IFN-γ secretion in human EBV-immortalized lymphoblastoid cell lines. (A) Cytotoxicity of resting and mrIL-15 (100ng/ml) activated splenocytes depicting killing of ^51^Cr-labeled YAC-1 targets from control (C) and *Clec16a* KO mice. Results include means±SE of five experiments (n = 5). (B) Interferon-γ release in T1D EBV cell lines measured by ELISA. EBV-immortalized lymphoblastoid cell lines generated from Type1 diabetes patients were activated with 100 ng/ml PMA plus 1 **μ**g/ml ionomycin (PMA+I). Culture supernatants were collected after 48 hrs of induction and IFN-γ was measured. “G/G” represents non-protective allele and “A/A” represents the protective allele for Type 1 diabetes. Results are means±SE of three independent repeats. C vs. KO (*P<0.05, **P<0.01, ***P<0.001), C vs. C+IL-15 (*P<0.05, **P<0.01, ***P<0.001), KO vs. KO+IL-15 (^#^P<0.05, ^##^P<0.01, ^###^P<0.001), C+IL-15 vs. KO+IL-15 (^$^P<0.05, ^$ $^P<0.01) by two-way ANOVA with Tukey’s multiple comparison test.

Given that NK cells constitute 1% of the total spleen immune cell population, and that the decrease we observed occurred in the total splenocyte numbers in KO mice, we performed functional evaluation of NK cells in *Clec16a* KO mice. We examined NK cytotoxicity in splenocytes isolated from *Clec16a* KO mice and controls with and without 48-hour rmIL-15 activation in a standard 4-hour ^51^Cr release assay. As expected, resting control splenocytes exhibited minimal killing of YAC-1 targets (7.8±1.8%; at 50:1 Effector: Target). By contrast, resting murine NK cells from *Clec16a* KO mice demonstrated increased killing (14.5±1.8%), a two-fold significant increase in cytotoxicity that remained significant at all effector: target ratios as compared to controls (Fig 4A). Cytotoxicity increased after IL-15 mediated activation in both the groups. At identical effector: target ratios, activated-control splenocytes demonstrated 30±2.5% cytotoxicity. *Clec16a* KO mice exhibited increase in cytotoxicity, with 45±2.7% that remained significant at all effector: target ratios. Thus, *Clec16a* knockout results in increased NK cytotoxicity in *Clec16a* KO mice.

We previously reported that the minor allele (A) at SNP rs2903692 is protective against T1D and associates with higher relative abundance of *CLEC16A* mRNA in NK cells. We predicted that possessing this allele will result in restrained NK cell functions. To test this, we evaluated EBV cell lines generated from type-1 diabetes patients homozygous for protective alleles (A/A) with higher levels of CLEC16A for IFN-γ production compared to the G/G homozygotes. As anticipated, the EBV cell lines generated from type-1 diabetes patients homozygous for protective alleles (A/A) with higher levels of CLEC16A show significantly reduced IFN-γ production compared to the G/G homozygotes (Fig 4B). Collectively, these results demonstrate that under normal conditions, CLEC16A plays a critical role in shaping the initial pro-inflammatory response possibly by constraining major NK cell functions.

**Fig 5:**
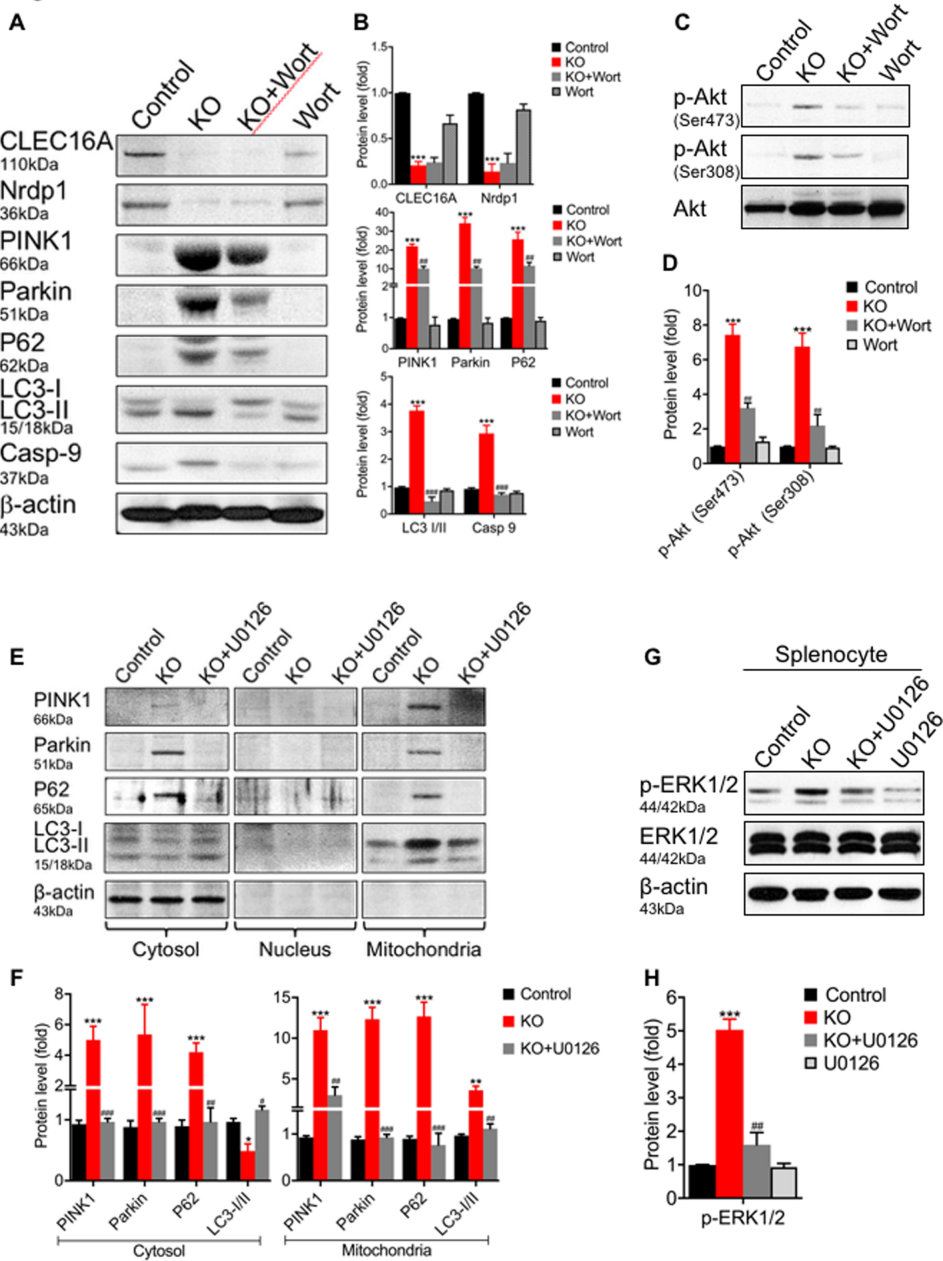
Wortmannin and U0126 reverse the disrupted mitophagy in *Clec16a* KO mice. (**A**) Representative western blot from splenocyte lysates of Control±Wortmannin and KO±Wortmannin treated mice depicting expression levels of CLEC16A, Nrdp1, PINK1, Parkin, P62, LC3 I/II, caspase-9 and rescue of disrupted by Wortmannin. Membranes were striped and reprobed for βactin as a loading control. (B) Quantitation graph depicting expression levels of CLEC16A, Nrdp1, PINK1, Parkin, P62, LC3I/II and Caspse-9 normalized to β-actin. Data expressed as means±SE of three independent experiments. (C) Representative western blot image of p-Akt (Ser473 or Ser308) and total Akt. (D) Quantitation graph depicting expression levels of p-Akt (Ser473 or Ser308). (E) Representative immunoblot from fractionated lysates (cytosol, nucleus and mitochondria fraction) of control (Vehicle), *Clec16a* KO and *Clec16a* KO+U0126, depicting expression levels of Nrdp1, PINK1, Parkin, P62, LC3 I/II and rescue of disrupted mitophagy by U0126. βactin was probed as loading control for cytosolic fraction. No β-actin was detected in nuclear and mitochondrial fraction. The cropped blots are part of the same gel separated by molecular weight marker. (G) Representative immunoblot image of p-ERK1/2, ERK1/2 and βactin in whole splenocyte cell lysate from control±U0126 and KO±U0126. Membranes were striped and reprobed for βactin as a loading control. (H) Graph of quantitation depicting p-ERK1/2. Data expressed as means±SE of three independent experiments. *P<0.05, **P<0.01, ***P<0.001, ^#^P<0.05, ^##^P<0.01, ^###^P<0.001. Significances of differences among groups were evaluated using unpaired Student’s t-test.

In light of enhanced intrinsic cell death and mitophagy defect observed above experiments were designed to determine whether PI3K inhibitor-Wortmannin and MEK inihibitor-U0126 could rescue the disrupted mitophagy observed in *Clec16a* KO mice. We treated mice with Wortmannin or U0126 and interrogated the mitophagy pathway in an immunoblot analysis for rescue in whole splenocytes lysates. In *Clec16a* KO, Nrdp1 expression significantly decreased in comparison to control (Fig 5A). Wortmannin alone had no effect on Nrdp1, PINK1, Parkin, P62, LC3-I/II and caspase-9 protein expression. The small decrease observed in CLEC16A expression with Wortmannin is unclear if this effect is related to Nrdp1 and could suggest a Nrdp1-independent role of CLEC16A. Parkin, P62, and Casp-9, and LC3-II showed significant increase in expression in *Clec16a* KO mice compared to control. Wortmannin-treated *Clec16a* KO mice showed a significant reversal in expression of PINK1, Parkin, P62, LC-3I/II and caspase-9 in comparison to KO (Fig 5A). To further confirm the specificity and involvement of PI3K signaling, we probed for p-Akt and total Akt in control±Wortmannin and *Clec16a* KO±Wortmannin treated mice. In *Clec16a* KO, we observed significant increase in p-Akt levels in comparison to control (Fig 5C and 5D). Wortmannin treatment significantly decreased the Akt phosphorylation in keeping with induced activation of Akt signaling in *Clec16a* KO mice.

We next evaluated rescue of disrupted mitophagy with the MEK inhibitor, U0126. We undertook a cell fractionation approach and performed immunoblot analysis on cytosol, nuclear and mitochondrial fraction (Fig 5E). The cytosolic fraction from *Clec16a* KO splenocytes showed detectable levels of PINK1, Parkin and p62. The mitochondrial fraction in KO showed significant up-regulation of PINK1, Parkin, P62 and LC3-II. U0126 treatment significantly abolished the expression of PINK1, Parkin, and P62 and reverted the LC3-II expression similar to control mice. PINK, Parkin and P62 were barely detectable in the cytosol and mitochondrial fractions of control mice. PINK1, Parkin, P62 and LC3-I/II were not detected in nuclear fractions of any mice (Fig 5E). U0126 inhibits MEK1 and MEK2, and thus ERK activation. To confirm the signaling involved and specificity of inhibitor, we probed splenocytes lysate for p-ERK and total ERK in control (Vehicle), *Clec16a* KO and *Clec16a* KO+U0126 treated mouse. As anticipated, in *Clec16a* KO, we observed significant increase in the levels of phosphorylated ERK 1/2 in comparison to control vehicle treated mice (Fig 5G and 5H). U0126 treated *Clec16a* KO mice showed significantly decreased ERK1/2 phosphorylation to basal level, suggesting the activation of ERK1/2 signaling in *Clec16a* KO with disrupted mitophagy. Control treated U0126 mice showed no significant increase or decrease in protein expression compared to control alone (S7A and S7B Fig). Thus, *Clec16a* knockout disrupts mitophagy in splenic immune cells. Taken together, these results suggest that Wortmannin and U0126, are capable of rescuing the disrupted mitophagy signaling defect in *Clec16a* KO mice.

**Fig 6:**
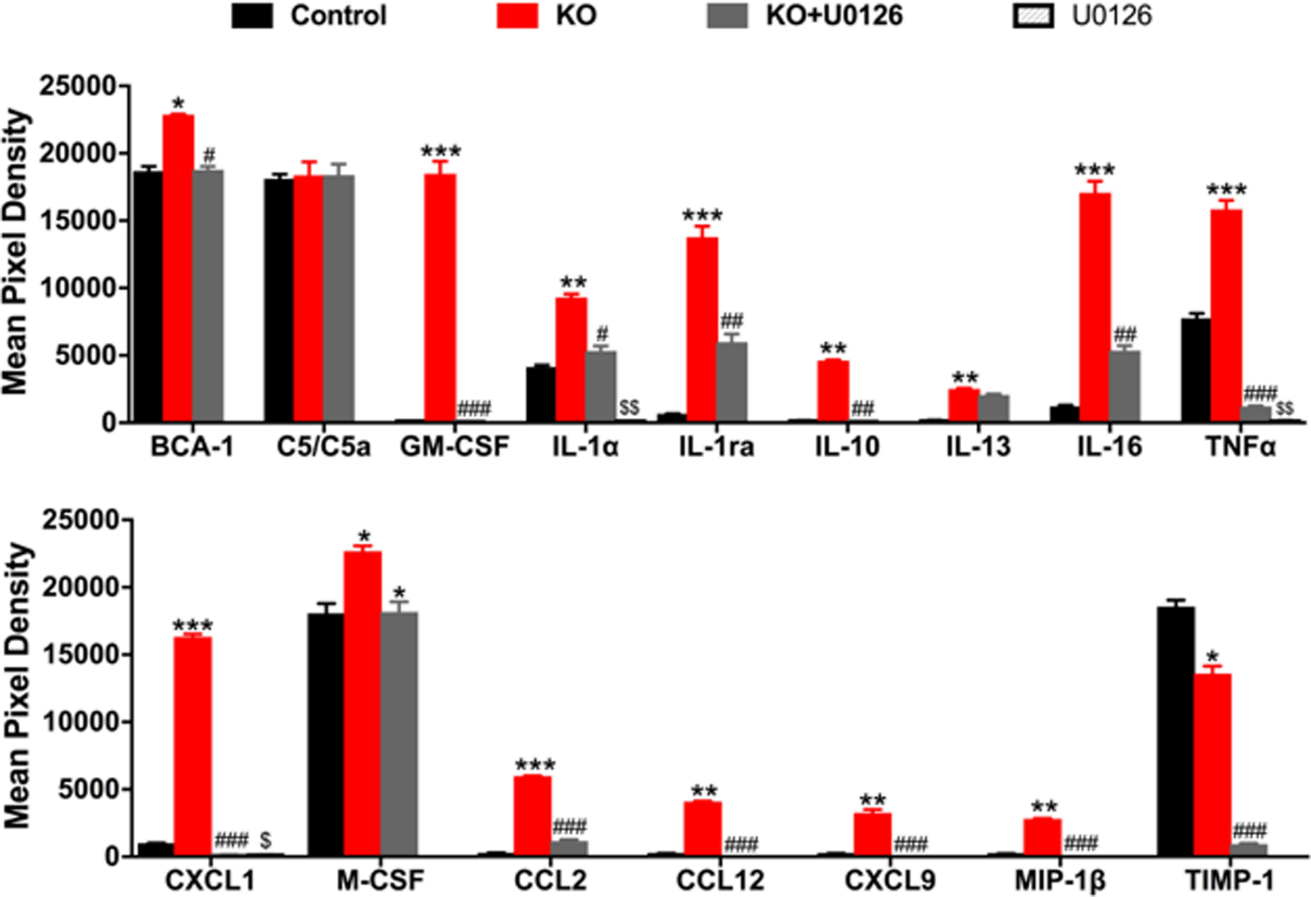
Predominant Th-1 Cytokine/chemokine in *Clec16a* KO and rescue with U0126. The representative graph is the quantification of cytokines and chemokine from plasma of Control (Vehicle), Control (U0126), KO and KO+U0126 inhibitor treated mice using the Mouse Cytokine Array panel. The amount of BCA-1 (CXCL13), C5/C5a, GM-CSF, IL-1α, IL-1ra, IL-10, IL-13, IL-16, KC (CXCL1), MCP-1, MCP-5, MIG (CXCL9), MIP-1β (CCL4), TIMP-1 and TNF-αin the plasma of control, *Clec16a* KO and Clec16a KO+U0126, and control+U0126 are reported. The results are presented as average signal (pixel density) of the pair of duplicate spots representing each cytokine or chemokine analyzed using Image-J software. Data expressed as means±SE of three independent experiments. *P<0.05, **P<0.01, ***P<0.001 represents C vs. KO, P<0.05, ^##^P<0.01, ^###^P<0.001 KO vs. KO+U0126, and ^$^P<0.05, ^$ $^P<0.01 C vs. U0126. Significances of differences among groups were evaluated using unpaired Student’s t-test.

Increased cytokines/chemokine levels reflect the inflammatory mechanism utilized during the development, progression and pathogenesis of various autoimmune and inflammatory diseases. To gain insight in the inflammatory mechanism we profiled plasma from control±U0126 and KO±U0126 treated mice for cytokines and chemokines using Mouse Cytokine Array panel (S8A and S8B Fig). Plasma from *Clec16a* KO mice showed more robust significant upregulation of Th1 cytokines (TNF-α, IL-1, & IL-16), vs. Th2 (IL-10 & IL-13) and elevated levels of key chemokines (GM-CSF, KC (CXCL1), M-CSF, MCP-1(CCL2), MCP-5 (CCL12), MIG (CXCL9), MIP-1β in comparison to control mice (Fig 6). U0126 treatment in *Clec16a* KO mice significantly reversed all the up-regulated cytokines and chemokines. U0126 treatment in control significantly reduced basal expression of IL-1α, CXCL1 and TNF-α. These results suggest the inflammatory mechanism involved in autoimmune diseases is mediated by disrupted mitophagy and can be attenuated in part by ERK1/2 inhibitor therapy.

## Discussion

*CLEC16A* is a well-established T1D susceptibility gene[1–5] and has also been convincingly implicated in MS[6, 7], PAI[8], CD[9], PBC[10], JIA[12], RA[12, 20], and AA[13]. More recently, CLEC16A has become an attractive candidate for studies addressing potential therapeutic targets. Despite this interest, little is known about CLEC16A function in humans. We previously reported in mice with pancreas-specific deletion of *Clec16a* that CLEC16A is required for normal glucose-stimulated insulin release through its effect on mitophagy[16]. The clinical significance of this work is supported by findings in patients with the *CLEC16A* T1D risk variant, rs12708716-G, who have reduced expression of CLEC16A in islets and attenuated insulin secretion. This work, however, does not address the complete role of CLEC16A in immune cell function, a critical component of autoimmunity.

Recent studies suggest that *CLEC16A* may control the HLA-II antigen presentation pathway via late endosomal maturation in antigen-presenting cells (APC)[21] and that *CLEC16A* variation impacts thymic selection playing a role in thymic epithelial cell (TEC) autophagy[22]. These data suggest that an increase in CLEC16A is provocative for autoimmune disease rather than a decrease in CLEC16A; that is, the previously proposed mechanism is at odds with the effect of the autoimmune risk variant, rs12708716-G, which results in reduced expression of CLEC16A[16]. By contrast, our work illustrates that decrease in CLEC16A expression via genetic *Clec16a* KO in mice led to disrupted/incomplete mitophagy, cell death and immune dysfunction.

To our knowledge, this is the first study elucidating the role of CLEC16A action in immune cells. We employed UBC-Cre-Clec16a^loxP^ mice—an inducible *Clec16a* KO model to study CLEC16A’s role on a whole organism level. We chose this model to circumvent possible embryonic lethality and determine the effect of CLEC16A loss on immune cells in adult mice.

Our whole body *Clec16a* KO mice exhibit atrophy of the thymus and spleen and rapid weight loss with no decrease in food intake. The atrophy observed in thymus is acute and in spleen is gradual. Spleen in KO mice showed reduced size and total splenocyte numbers suggesting increased cell death. Splenic immune cells of *Clec16a* KO mice exhibit lowered mitochondrial membrane potential. TEM images reveal changes in the mitochondrial membrane architecture and accumulation of disordered, disintegrated, elongated, and fragmented un-healthy mitochondria in splenic immune cells. Our data shows that lowered mitochondrial membrane potential initiates Nrdp1/PINK/Parkin1 dependent, incomplete mitophagy processing and activation of intrinsic pathway of cell death if impaired mitochondria are not disposed effectively.

Autophagic flux was reduced in splenocytes suggesting incomplete mitophagy in *Clec16a* KO mice as indicated by modest increase in LC-3 I/II and lack of change in P62 with Bafilomycin treatment. P62 protein is itself degraded by autophagy and serves as a marker to study autophagic flux. Pure B and T-cells in *Clec16a* KO revealed the same reduced autophagic flux as indicated by accumulation of P62. Our whole-body inducible *Clec16a* KO murine model demonstrates that post-developmental *Clec16a* knockout results in increased NK cytotoxicity in *Clec16a* KO mice independent of NK cell activation, consistent with immune dysfunction. Hyperactive NK cell may be most influential at the initiation of the autoimmune response through interactions with T and B cells. To our knowledge, no one has shown the link between CLEC16A expression and immune dysfunction. Failure to remove dysfunctional mitochondria leads to hyper-activation of inflammatory pathways and enhancement of the inflammatory function in major auto-inflammatory and autoimmune diseases[23]. Dead cells constitute a source of novel antigens and proinflammatory molecules that can provoke autoimmune response. Exposure of novel antigens to the immune system may be the lead trigger of abnormal immune response caused by polarized innate immune cells and other cells of the local environment. As a consequence, the resulting activation and polarization of innate and adaptive immune cells towards a Th-1 type immune response may be among key factors contributing to the pathogenesis. This is further supported by the fact that conditional targeting of T cells in CD4 Cre Clec16a^loxP^ mice showed no difference in T cell repertoire and pathological phenotype.

Malfunctioning autophagy, either too little or too much can be detrimental to cells. Harmful imbalances in autophagic regulation are conceptualized as a state of autophagic stress in many human diseases including cancer, neurodegenerative, cardiac, infectious, inflammatory and autoimmune diseases[24–27]. Autophagy and apoptosis are important and interconnected stress response mechanisms. The effect of autophagy on disease progression has not yet been discovered, and the identification and development of new drug targets is still a key focus. Many lines of evidence support the existence of cross-talk between PI3K and ERK and the potential for PI3K to act as an upstream activator of ERK [28, 29]. ERK activity has been associated with classical markers of apoptosis execution, such as effector caspase-3 activation, characterized by release of cytochrome-c from mitochondria and activation of initiator caspase-9. Inhibition of autophagy leads to enhanced cell death and inflammation as observed in our *Clec16a* KO mice. PI3K inhibitor-Wortmannin and MEK inihibitor-U0126 attenuated the mitophagy defects observed in the *Clec16a* KO murine model and provide evidence in support of the future therapies that depend on ability to correct disease related factors that promote autophagic stress and contribute to pathological imbalances in the system.

Our study underscores a critical role of CLEC16A action in immune cells signaling through the Nrdp1/PINK/Parkin pathway. A delicate balance of CLEC16A activity appears to be needed for cellular homeostasis. In patient populations harboring variants that result in CLEC16A hypofunction, drugs with modulatory effects on mitophagy could compensate for the attenuated CLEC16A activity and present formidable candidates for targeted interventions.

## Supporting information

S1 Fig Generation of *Clec16a* KO UBC-*Cre-Clec16a*^loxP/loxP^ mice. Schematic representation of Cre-mediated recombination of the *Clec16a* locus.
(TIFF)

S2 Fig *Clec16a* knockout disrupts mitophagy *in-vivo* in response to lowered mitochondrial membrane potential resulting in aggregation of fragmented mitochondria in splenic immune cells. (A) Representative histogram depicts the basal mitochondrial membrane potential in control and *Clec16a* KO splenocytes at 24 and 48hrs without stimulation using MitoTracker Deep Red FM dye in a flow-based assay. (B) Mitochondrial membrane potential depicted as change in mean fluorescence intensity for two time points, 24h and 48h in unstimulated control and *Clec16a* KO splenocytes. (C) Immunoblot depicts CLEC16A expression in control and *Clec16a* KO splenocytes at 24 and 48hrs. (D) Representative immune blot image from control±IL-15 and KO±IL-15 splenocyte lysate depicting levels of P62 and LC3-I/II. (E) Quantitation graph depicting expression levels of P62 and LC3-I/II. (F) Representative immune blot images from splenocyte lysate depicting CLEC16A Nrdp1, PINK1, Parkin, P62, TOM20, ATG16L1, LC3I/II, Cytochrome-c, and Caspase-9 expression in Control±Bafilomycin treated mice. (G) Quantitation graph depicting expression levels of CLEC16A, Nrdp1, PINK1, Parkin, P62, TOM20, ATG16L1, LC3I/II, Cytochrome-c, and Caspase-9 and cleaved caspase-3 from Control±Bafilomycin treated mice normalized to β-actin. Data expressed as means±SE of three independent experiments. *P<0.05, **P<0.01, ***P<0.001. ^#^P<0.05, ^##^P<0.01.
(TIFF)

S3 Fig TEM image of B cell from *Clec16a* KO. Zoomed in TEM image of B-cell from *Clec16a* KO mice depicts mitochondria with abnormal morphology (white arrows).
(TIFF)

S4 Fig TEM image of T cell from *Clec16a* KO. Zoomed in TEM image of T-cell from *Clec16a* KO mice depicts mitochondria with abnormal morphology (white arrows).
(TIFF)

S5 Fig T cell subpopulation comparison of control and *Clec16a* KO mice. (A) The dot plot shows CD4 (top-panel) and CD8-T (bottom-panel) cell subpopulation between control (vehicle) and TAM-treated mice. (B) Graph depicts percent of CD4+ naïve, memory and effector T cells. (C) Graph depicts percent of CD8+ cells naïve, memory and effector T cells. Results for each group (Control n = 7; *Clec16a* KO n = 10) are presented as means±SE. **P<0.01, ***P<0.001 (unpaired two-tailed Student’s t-test). Splenocytes were stained with anti-CD3, anti-CD4, anti-CD8, anti-CD62L, anti-CD44, and UV live/dead. Cells were gated on CD3^+^CD4^+^ or CD3^+^CD8^+^ cells. Numbers indicate the percentage of Naïve and effector cells.
(TIFF)

S6 Fig CD4 Cre Clec16a^loxP^ mice T-cell subpopulation analysis. CD4 *Cre Clec16a*^loxP^ mice T-cell subpopulation analysis from thymus (A) and spleen (B) showed no significant difference. CD4 *Cre Clec16a*^loxP^ mice. *Clec16a*^loxP^ mice were mated to B6.Cg-Tg (CD4-cre) 1Cwi/BfluJ mice (The Jackson Laboratory). CD4-Cre transgenic mice contain CD4 enhancer, promoter and silencer sequences driving the expression of a Cre recombinase gene. By crossing *Clec16a*^loxP^ mice to this strain we generated CD4 Cre *Clec16a*^loxP^ mice with *Clec16a* conditional mutations in CD4-expressing tissues. Specifically, Cre recombinase expression is observed in CD4-expressing T cells during sequential stages of T cell development in lymphoid tissues. Mutation of the *Clec16a* gene was confirmed by PCR on DNAs and by RT-PCR on RNAs of T cells isolated from splenocytes of *Clec16a* CD4 T cell specific mutant and control littermate mice.
(TIFF)

S7 Fig Control±U0126 Immunoblot for mitophagy. (A) Representative immune blot images from splenocyte lysate depicting CLEC16A Nrdp1, PINK1, Parkin, P62, TOM20, ATG16L1, LC3I/II, Cytochrome-c, and Caspase-9 expression in Control±U0126 treated mice. (B) Quantitation graph depicting expression levels of CLEC16A, Nrdp1, PINK1, Parkin, P62, TOM20, ATG16L1, LC3I/II, Cytochrome-c, and Caspase-9 from Control±U0126 treated mice normalized to β-actin. Data is expressed as means±SE of three independent experiments.
(TIFF)

S8 Fig Mouse Cytokine Array. (A) Representative Array blot of plasma cytokine and chemokine from Control (Vehicle), Control (U0126), *Clec16a* KO, and U0126 treated *Clec16a* knockout mice. For each, 100 ul of plasma was run on the array. Data shown are from a two-hour exposure to X-ray film. The average signal (pixel density) of the pair of duplicate spots representing each cytokine or chemokine was analyzed using Image-J software (B). Table depicts cytokines, chemokines, adipokines, growth factors and immune related proteins coordinates on Mouse Cytokine Array Panel A.
(TIFF)
