# Supplementary figures and images for: CLEC16A regulates splenocyte and NK cell function in part through MEK signaling
Source: PLoS One. 2018 Sep 18;13(9):e0203952. doi: 10.1371/journal.pone.0203952 (PMC6143231; doi:10.1371/journal.pone.0203952)

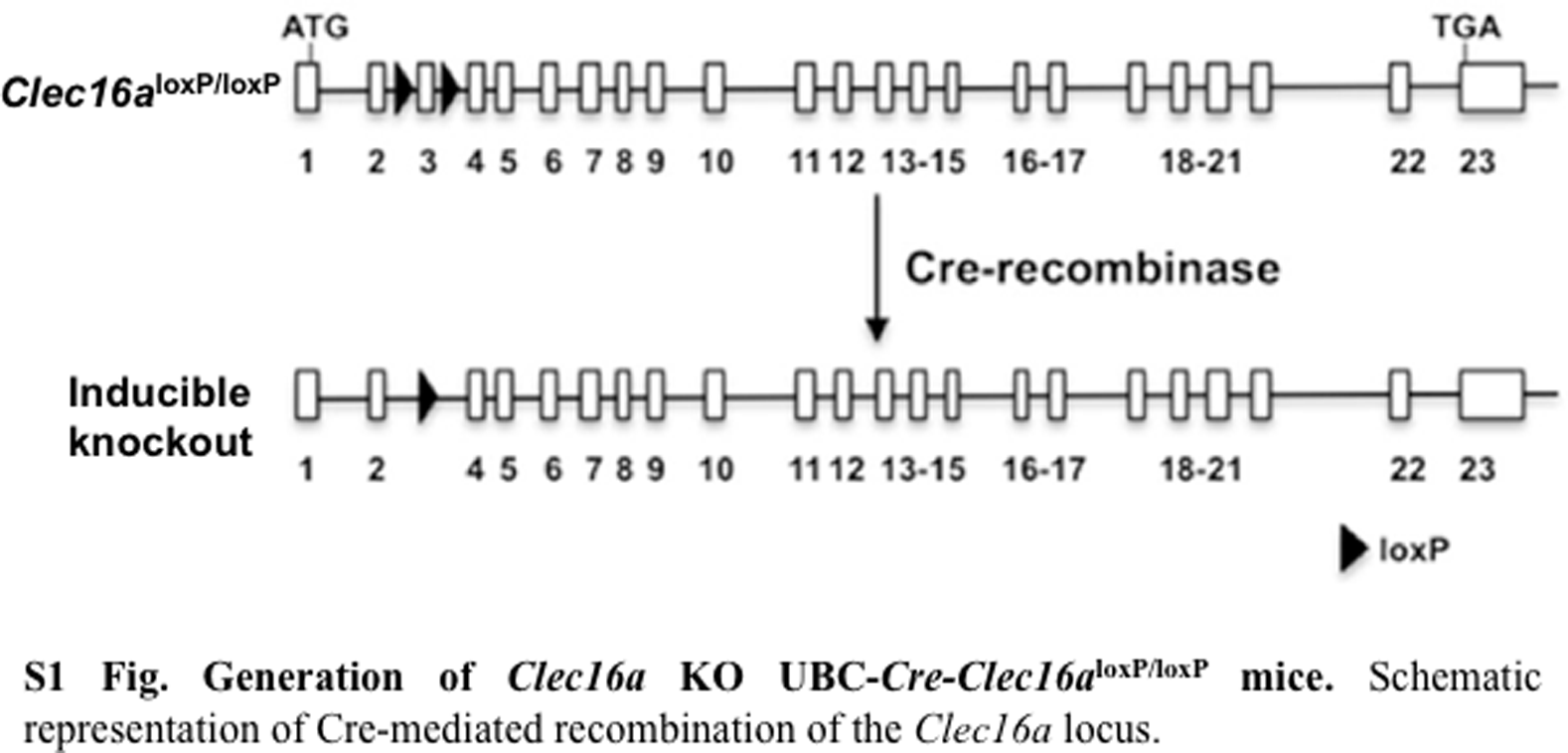

Supplement: S1 Fig — Schematic representation of Cre-mediated recombination of the Clec16a locus. (TIFF) [file pone.0203952.s001.tiff]

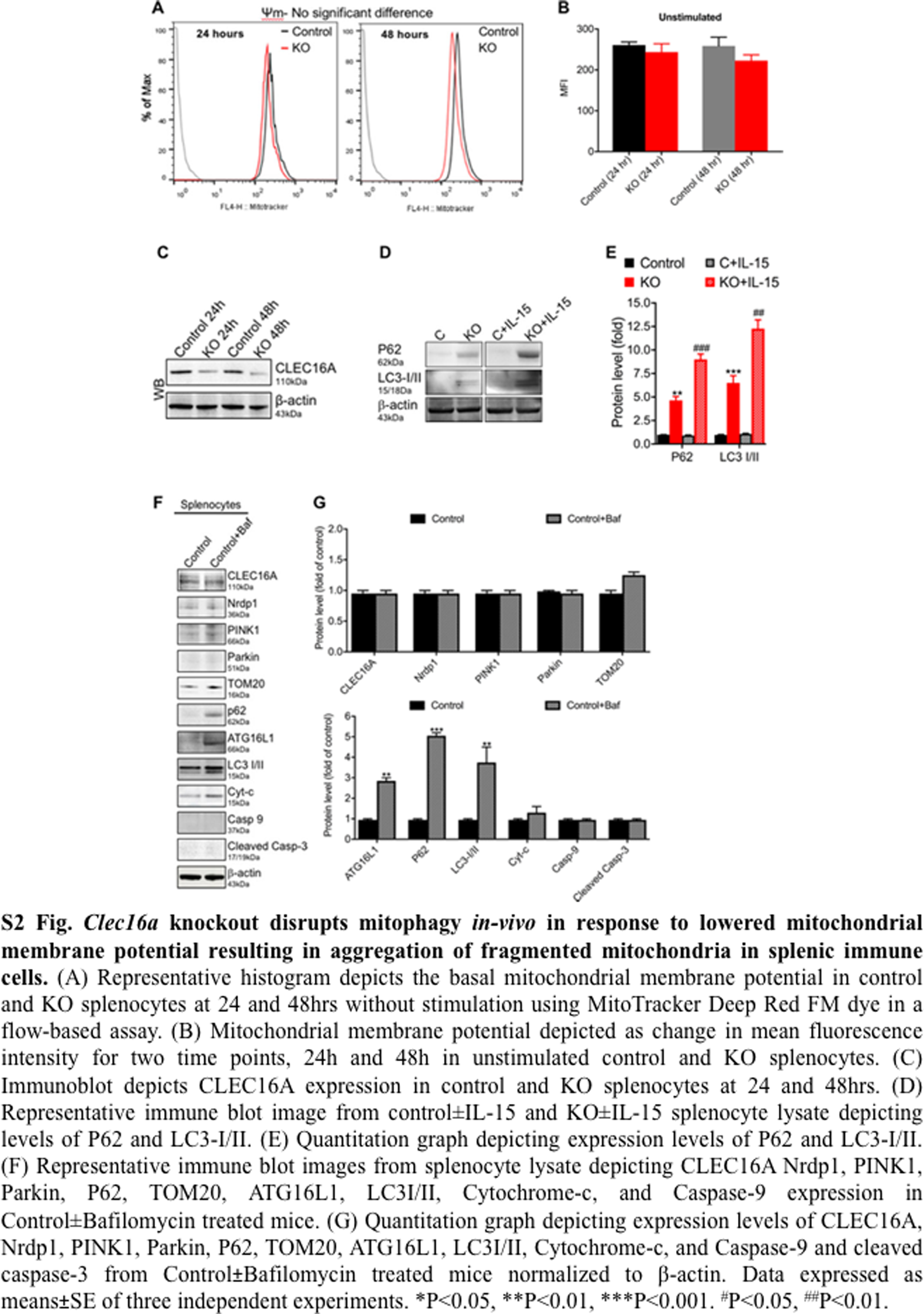

Supplement: S2 Fig — (A) Representative histogram depicts the basal mitochondrial membrane potential in control and Clec16a KO splenocytes at 24 and 48hrs without stimulation using MitoTracker Deep Red FM dye in a flow-based assay. (B) Mitochondrial membrane potential depicted as change in mean fluorescence intensity for two time points, 24h and 48h in unstimulated control and Clec16a KO splenocytes. (C) Immunoblot depicts CLEC16A expression in control and Clec16a KO splenocytes at 24 and 48hrs. (D) Representative immune blot image from control±IL-15 and KO±IL-15 splenocyte lysate depicting levels of P62 and LC3-I/II. (E) Quantitation graph depicting expression levels of P62 and LC3-I/II. (F) Representative immune blot images from splenocyte lysate depicting CLEC16A Nrdp1, PINK1, Parkin, P62, TOM20, ATG16L1, LC3I/II, Cytochrome-c, and Caspase-9 expression in Control±Bafilomycin treated mice. (G) Quantitation graph depicting expression levels of CLEC16A, Nrdp1, PINK1, Parkin, P62, TOM20, ATG16L1, LC3I/II, Cytochrome-c, and Caspase-9 and cleaved caspase-3 from Control±Bafilomycin treated mice normalized to β-actin. Data expressed as means±SE of three independent experiments. *P<0.05, **P<0.01, ***P<0.001. #P<0.05, ##P<0.01. (TIFF) [file pone.0203952.s002.tiff]

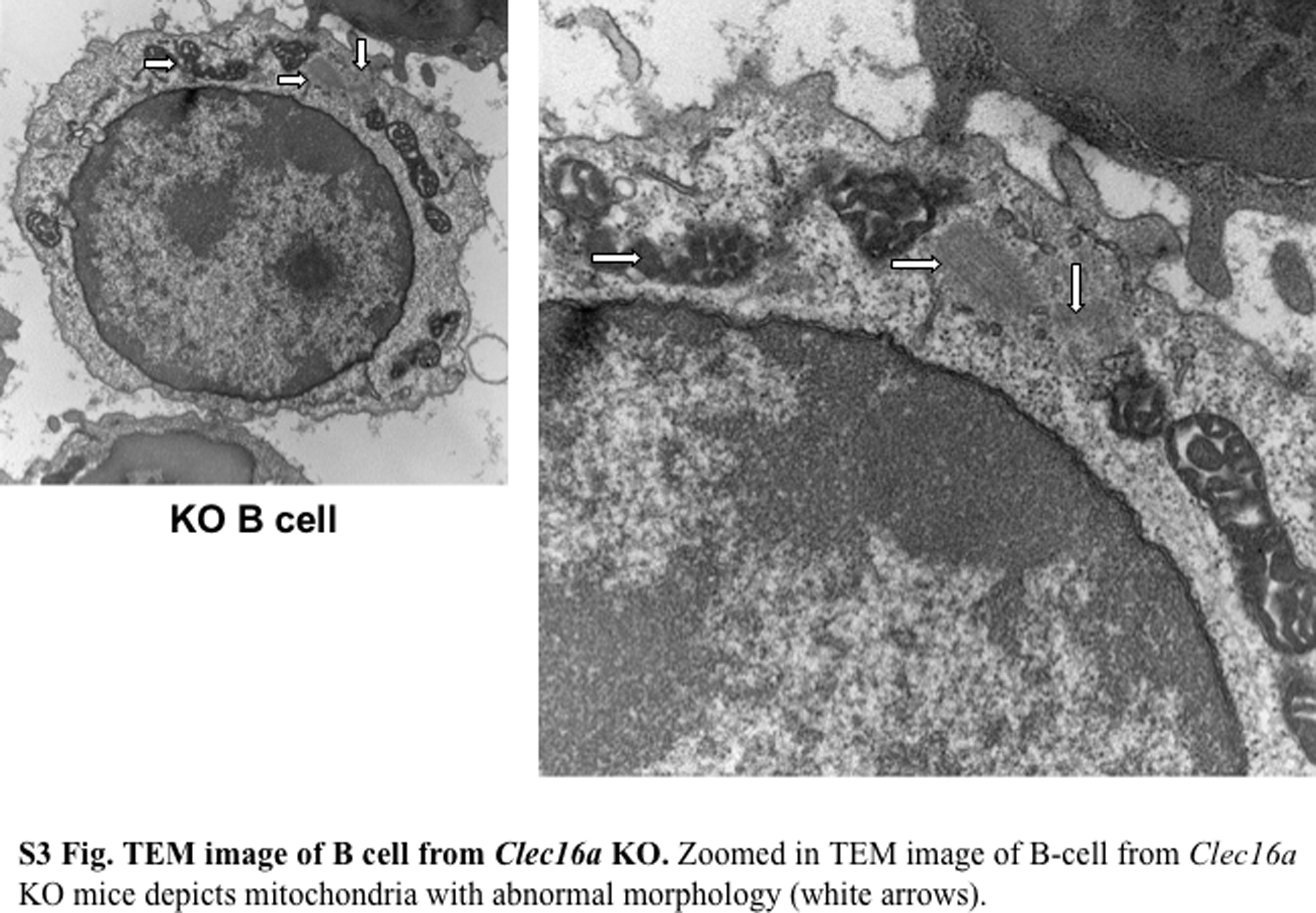

Supplement: S3 Fig — Zoomed in TEM image of B-cell from Clec16a KO mice depicts mitochondria with abnormal morphology (white arrows). (TIFF) [file pone.0203952.s003.tiff]

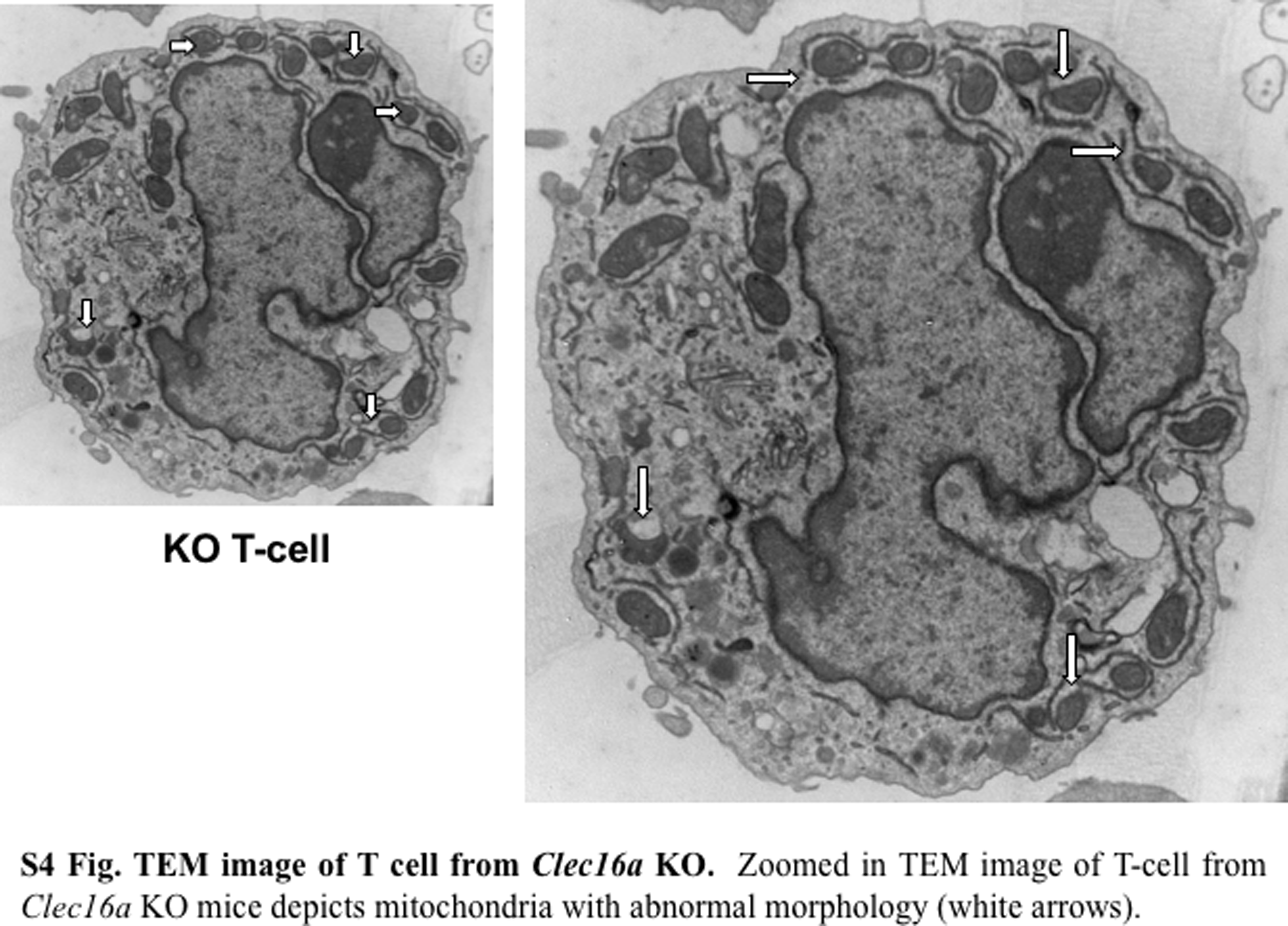

Supplement: S4 Fig — Zoomed in TEM image of T-cell from Clec16a KO mice depicts mitochondria with abnormal morphology (white arrows). (TIFF) [file pone.0203952.s004.tiff]

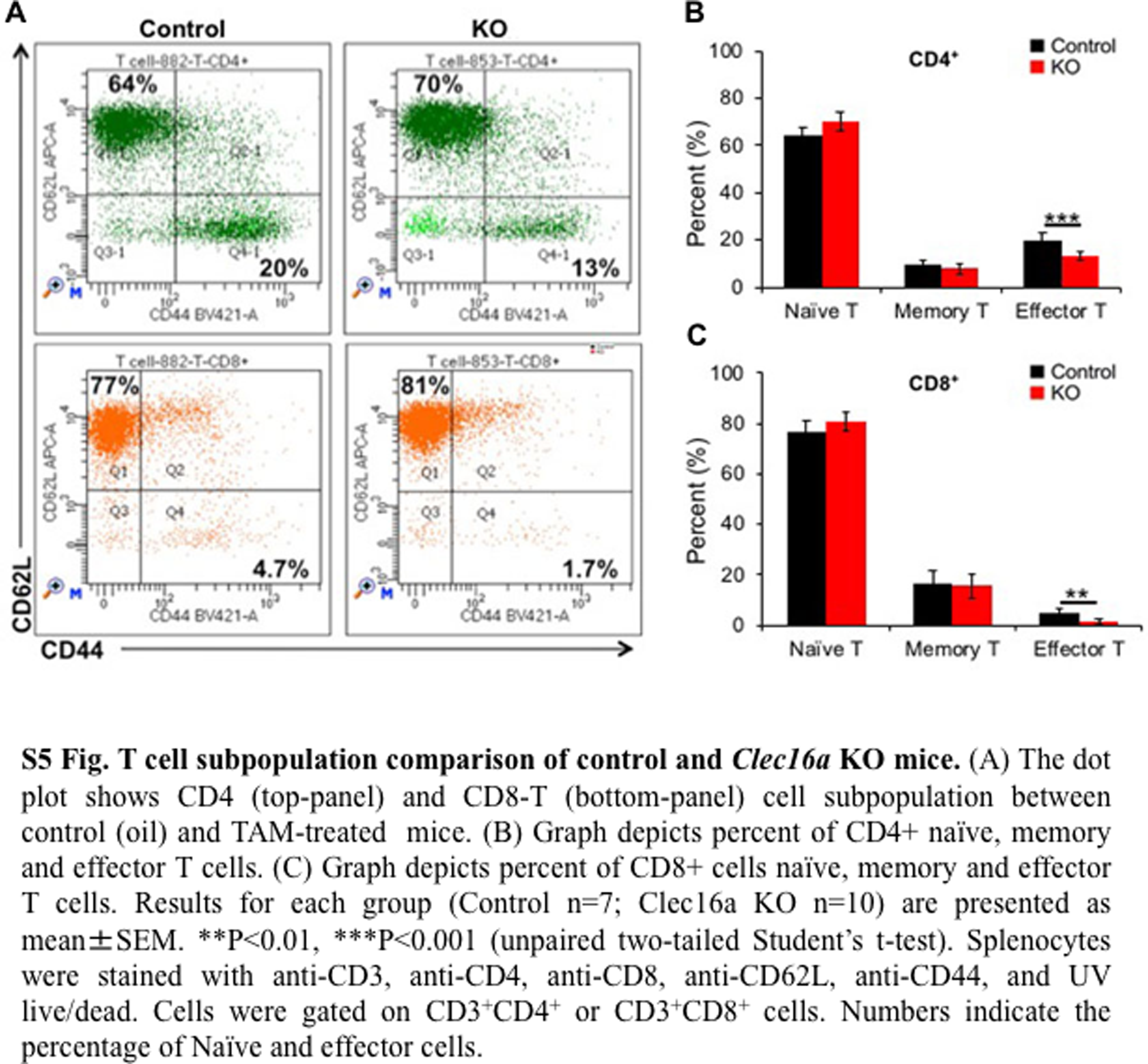

Supplement: S5 Fig — (A) The dot plot shows CD4 (top-panel) and CD8-T (bottom-panel) cell subpopulation between control (vehicle) and TAM-treated mice. (B) Graph depicts percent of CD4+ naïve, memory and effector T cells. (C) Graph depicts percent of CD8+ cells naïve, memory and effector T cells. Results for each group (Control n = 7; Clec16a KO n = 10) are presented as means±SE. **P<0.01, ***P<0.001 (unpaired two-tailed Student’s t-test). Splenocytes were stained with anti-CD3, anti-CD4, anti-CD8, anti-CD62L, anti-CD44, and UV live/dead. Cells were gated on CD3+CD4+ or CD3+CD8+ cells. Numbers indicate the percentage of Naïve and effector cells. (TIFF) [file pone.0203952.s005.tiff]

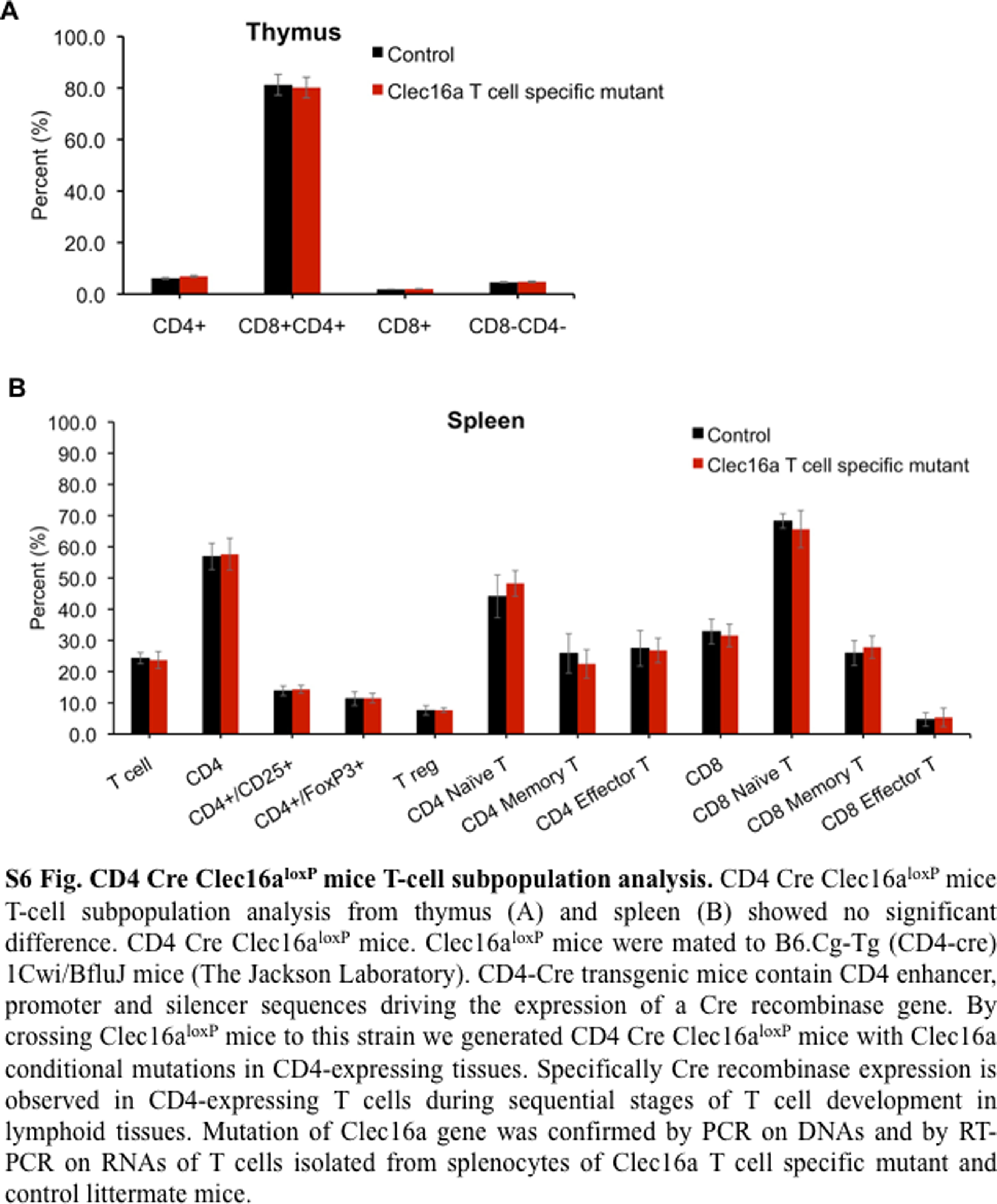

Supplement: S6 Fig — CD4 Cre Clec16aloxP mice T-cell subpopulation analysis from thymus (A) and spleen (B) showed no significant difference. CD4 Cre Clec16aloxP mice. Clec16aloxP mice were mated to B6.Cg-Tg (CD4-cre) 1Cwi/BfluJ mice (The Jackson Laboratory). CD4-Cre transgenic mice contain CD4 enhancer, promoter and silencer sequences driving the expression of a Cre recombinase gene. By crossing Clec16aloxP mice to this strain we generated CD4 Cre Clec16aloxP mice with Clec16a conditional mutations in CD4-expressing tissues. Specifically, Cre recombinase expression is observed in CD4-expressing T cells during sequential stages of T cell development in lymphoid tissues. Mutation of the Clec16a gene was confirmed by PCR on DNAs and by RT-PCR on RNAs of T cells isolated from splenocytes of Clec16a CD4 T cell specific mutant and control littermate mice. (TIFF) [file pone.0203952.s006.tiff]

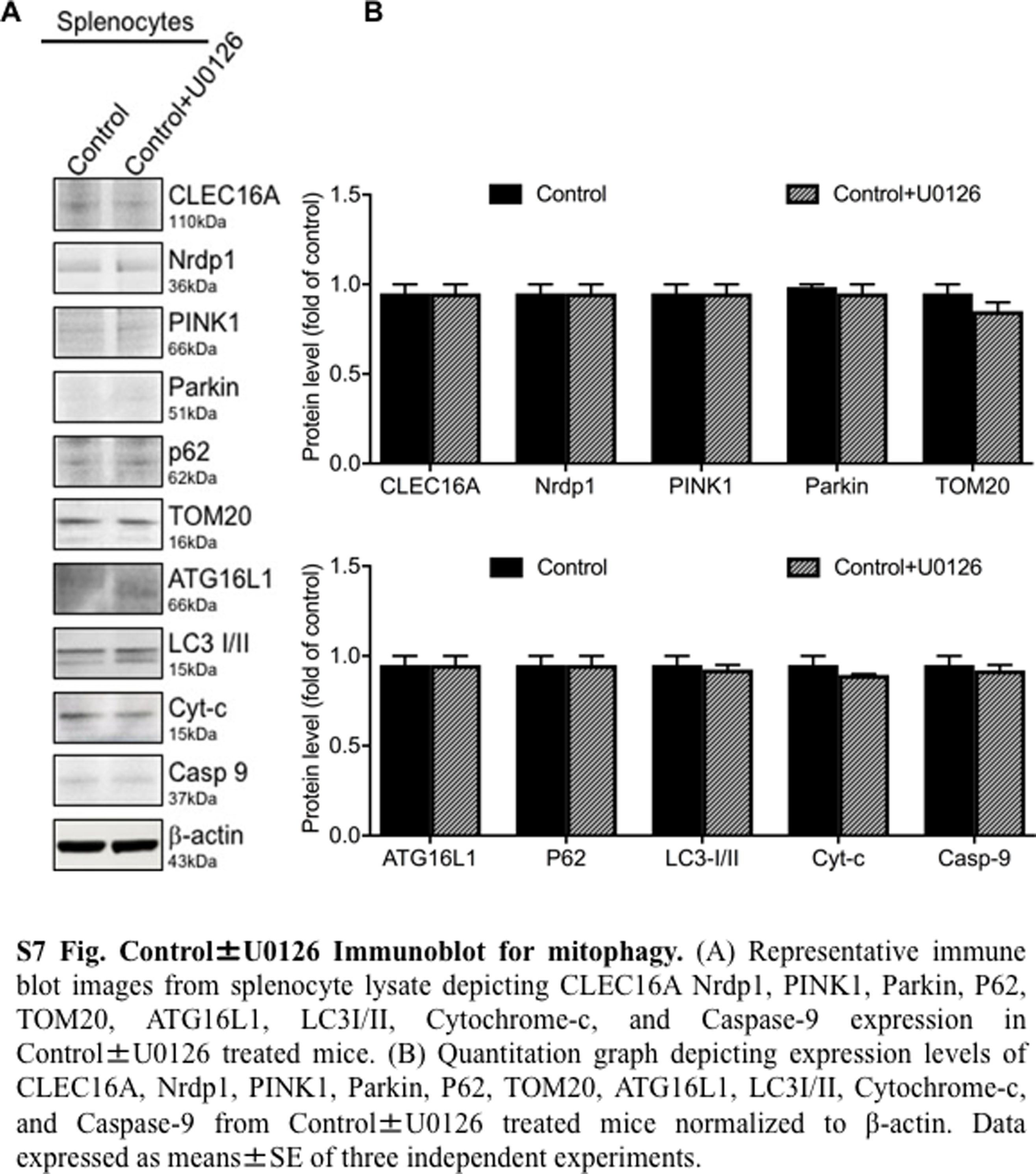

Supplement: S7 Fig — (A) Representative immune blot images from splenocyte lysate depicting CLEC16A Nrdp1, PINK1, Parkin, P62, TOM20, ATG16L1, LC3I/II, Cytochrome-c, and Caspase-9 expression in Control±U0126 treated mice. (B) Quantitation graph depicting expression levels of CLEC16A, Nrdp1, PINK1, Parkin, P62, TOM20, ATG16L1, LC3I/II, Cytochrome-c, and Caspase-9 from Control±U0126 treated mice normalized to β-actin. Data is expressed as means±SE of three independent experiments. (TIFF) [file pone.0203952.s007.tiff]

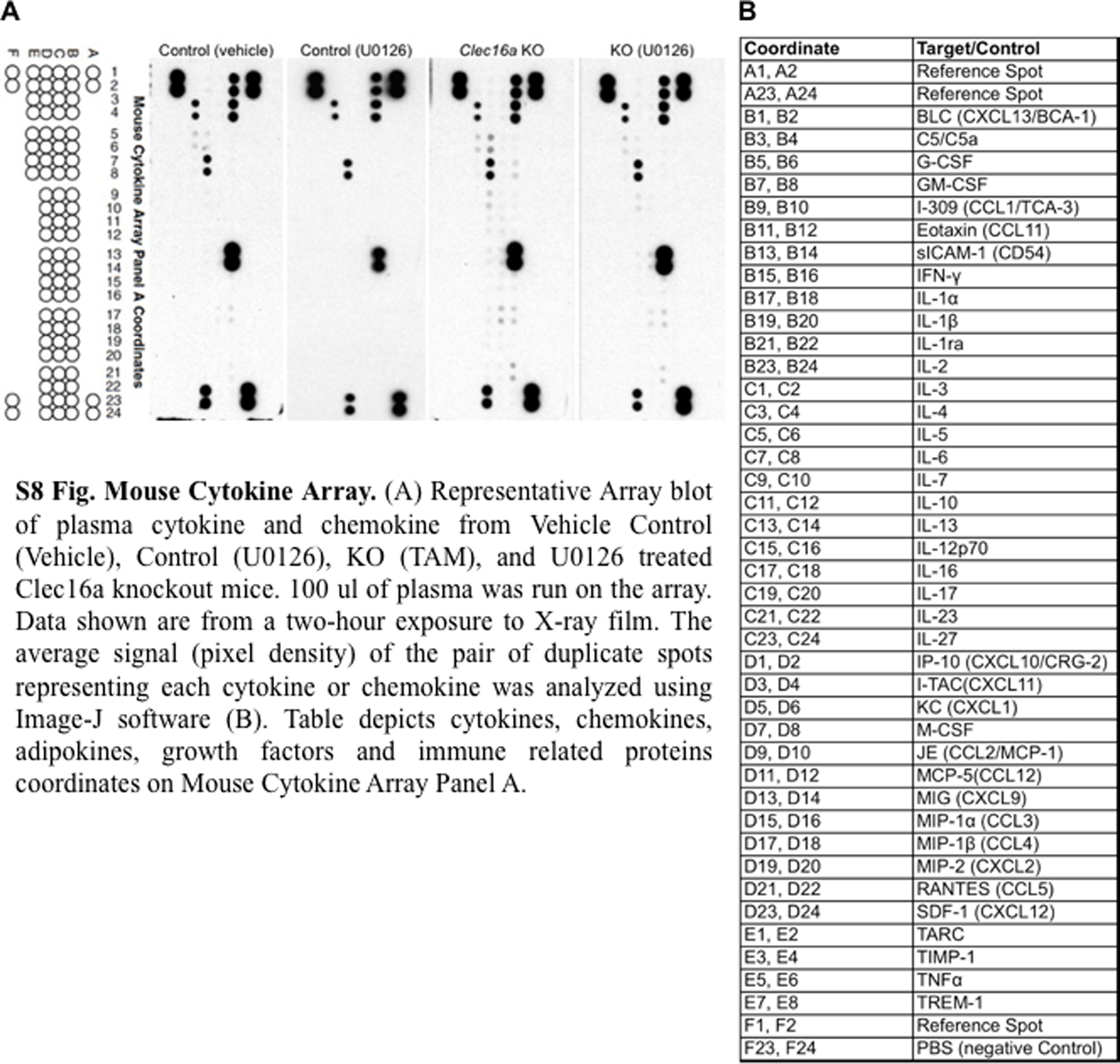

Supplement: S8 Fig — (A) Representative Array blot of plasma cytokine and chemokine from Control (Vehicle), Control (U0126), Clec16a KO, and U0126 treated Clec16a knockout mice. For each, 100 ul of plasma was run on the array. Data shown are from a two-hour exposure to X-ray film. The average signal (pixel density) of the pair of duplicate spots representing each cytokine or chemokine was analyzed using Image-J software (B). Table depicts cytokines, chemokines, adipokines, growth factors and immune related proteins coordinates on Mouse Cytokine Array Panel A. (TIFF) [file pone.0203952.s008.tiff]
